# Development of maximum relevant prior feature ensemble (MRPFE) index to characterize future drought using global climate models

**DOI:** 10.1038/s41598-024-66804-5

**Published:** 2024-07-09

**Authors:** Atta Gul, Sadia Qamar, Mahrukh Yousaf, Zulfiqar Ali, Mohammed Alshahrani, Shreefa O. Hilali

**Affiliations:** 1 Department of Statistics, University of Sargodha, Sargodha, Pakistan; 2 College of Statistical Sciences, University of the Punjab, Lahore, Pakistan; 3 Department of Mathematics, College of Sciences and Humanities, Prince Sattam Bin Abdulaziz University, 11942 Alkharj, Saudi Arabia; 4 Department of Mathematics, College of Sciences and Arts (Majardah), King Khalid University, 61937 Magardah, Saudi Arabia

**Keywords:** Drought, Global warming, Multiple global climate models (GCMs), Coupled model intercomparison project Phase 6 (CMIP6), Climate sciences, Hydrology, Natural hazards

## Abstract

Drought is one of the foremost outcomes of global warming and global climate change. It is a serious threat to humans and other living beings. To reduce the adverse impact of drought, mitigation strategies as well as sound projections of extreme events are essential. This research aims to strengthen the robustness of anticipated twenty-first century drought by combining different Global Climate Models (GCMs). In this article, we develop a new drought index, named Maximum Relevant Prior Feature Ensemble index that is based on the newly proposed weighting scheme, called weighted ensemble (WE). In the application, this study considers 32 randomly scattered grid points within the Tibetan Plateau region and 18 GCMs of Coupled Model Intercomparison Project Phase 6 (CMIP6) of precipitation. In this study, the comparative inferences of the WE scheme are made with the traditional simple model averaging (SMA). To investigate the trend and long-term probability of various classes, this research employs Markov chain steady states probability, Mann–Kendall trend test, and Sen’s Slope estimator. The outcomes of this research are twofold. Firstly, the comparative inference shows that the proposed weighting scheme has greater efficiency than SMA to conflate GCMs. Secondly, the research indicates that the Tibetan Plateau is projected to experience “moderate drought (MD)” in the twenty-first century.

## Introduction

Global warming refers to the rapidly increasing temperature on the earth’s surface over a long period^1^. Global warming is mostly caused by rises in greenhouse gases, including methane (${CH}_{4}$), hydrogen (H), nitrous oxide (${N}_{2}$O), carbon dioxide (${CO}_{2}$), and sulfur dioxide (${SO}_{2}$). ${CO}_{2}$ emissions in the atmosphere from power plants, factories, vehicles, volcanic eruptions, and burning of coal and gas are the key issues of global warming^2^. It will affect the earth’s geology^3^, ocean, and atmosphere^4^. One of the adverse effects of global warming is that the reservoir of freshwater resources has been depleted at an unprecedented ratio. Pakistan is also experiencing the effects of global warming^5^. In several undeveloped areas, death rates have been reported in many parts of the country due to global warming^6^. Thousands of people died due to harsh conditions resulting from the scarcity of clean water in Tharparker, particularly in the 2010s^7^. This scarcity is closely related to climate change, which has made the region’s already dry conditions even worse. Significant reductions in rainfall lead to prolonged droughts in Tharparker. These droughts dried water sources, making it difficult for the local population to access clean drinking water^6^. Furthermore, many researchers noted the decreasing trend of rainfall in Pakistan attributable to climate change^8^. This trend has been observed over the past few decades^5,9^. Drought has also harmed many aspects of life in the majority of the world as a result of global warming^10^. Drought is an extreme event of climate that occurs when the precipitation is below than average level. The global agricultural sector faces severe drought risk due to rapid changes in the global warming environment. It is posing significant threats to pollution levels^11^, agriculture, livestock^12^, ecosystems, and human health^13^. Nevertheless, effective planning and drought moderation techniques can mitigate these adverse effects to some extent. Drought moderation techniques include methods like rainwater harvesting, building reservoirs and dams, and planting drought-resistant crops.

A Global Climate Model (GCM) is a mathematical depiction of the Earth’s atmosphere designed to simulate the Earth's climate system and ocean circulations^14,15^. It is essential for climate studies, as it enables the refinement of our comprehension and projecting capabilities regarding the atmosphere^16^, ocean, and climate behavior^17^. GCM simulations are commonly employed for assessing future drought risks. These GCM simulations have been utilized by various researchers, including^18–20^. Numerous researchers have utilized hydrological data to evaluate drought indices for estimating drought severity. Examples include the Palmer Drought Severity Index (PDSI)^21^, the Normalized Ecosystem Drought Index (NEDI)^22^, and the Standardized Precipitation Index (SPI)^23^. Similarly,^24^ also utilized precipitation data of GCMs to study future conditions of drought. GCM models exhibit significant biases, particularly for the variables influencing hydrology.

The assessment of climate variables in the Coupled Model Intercomparison Project phase 5 (CMIP5) and CMIP6 models is prone to a certain amount of uncertainty and fluctuation. CMIP5 and CMIP6 simulate Earth's climate system and are used to project future climate scenarios under different greenhouse gas emission scenarios^24^. Numerous authors have found that it is challenging to accurately estimate extreme hydrological occurrences because of the uncertainty of climate projection models^25^. Using a single climate model decreases the reliability of the results in analyzing meteorological events^26^. Numerous researchers suggested that using ensemble models might help to reduce the uncertainty^27,28^.

Drought has been evaluated in a range of climate simulation scenarios using the ensemble approach many times. For instance,^29^ employed statistical and machine-learning techniques to construct an ensemble approach for thirty-four CMIP5 climate models^30^. Utilized the multi-model ensemble and the delta method to project future temperature changes. Ruan et al.^31^ examined potential fluctuations in temperature, precipitation, and drought characteristics using the CMIP5 optimum ensemble of GCMs. However, biases and some estimation errors are inherent in every model ensemble, lowering the reliability of models^32^. Hence, to accurately estimate drought conditions, it is necessary to use methods that can project droughts efficiently. This ensures a more comprehensive understanding of drought dynamics. Therefore, this research aims to propose a comprehensive technique for studying future drought conditions for the time 2015–2100 at different time scales (i.e., 1, 3, 6, 9, 12, 24, and 48 months). Time scales help to evaluate which type of drought it is. Short-term precipitation deficits indicate meteorological drought, medium-term soil moisture deficits point to agricultural drought, and long-term reductions in water bodies signal hydrological drought. Socioeconomic drought can encompass various time scales, depending on its impact on society and the economy.

The resulting Maximum Relevant Prior Feature Ensemble (MRPFE) drought index allows for efficient and accurate drought estimations.

## Data and methods

The methods and study areas utilized in this study briefly explained in the following sub-sections. However,^32,33^ utilized the same study area for studying drought. Whereas, the standardization procedure is selected, based on^34^ research. Moreover,^24^ also studied the long-term statistic of precipitation by using Steady state probabilities.

### K-component Gaussian mixture distribution (K-CGMD)

The Standardized Drought Index (SDI) is a crucial measure for assessing drought severity. Fitting an appropriate probability distribution to time series precipitation data is a key point in measuring the SDI. The meteorological variables data follow a multimodal distribution, which means that the distribution of the data has more than one peak. The current SDI estimation methodology is based on an unimodal distribution. In such cases, insufficient distribution fitting reduces the accuracy of drought assessment. In past research, unimodal distributions were commonly employed to compute drought indices such as the Standardized Precipitation Index (SPI)^23^ and standardized Precipitation Evapotranspiration Index (SPEI)^35^. However, these indices are multimodal^5^. On another aspect, multimodal distributions can improve computational accuracy. The R packages *‘*fitdistrplus*’* and ‘propagate’ are used to select the appropriate probability function in this study. Recently,^36,37^ fitted 32 probability distributions for the calculation of various drought indices using the ‘R’ package. Ali et al.^5^ used K-CGMD based on a standardization method to model precipitation time series and achieve the highest computational accuracy. K-CGMD is a type of mixture model that has been used in a variety of studies to simulate various random events^24,37^. Mathematically, the K-CGMDs are presented as:

1 $$P(x) =\sum_{i=1}^{k}{w}_{i}N(y|{s}_{i},{q}_{i})$$

2 $$N\left(y|{s}_{i},{q}_{i}\right)=\frac{1}{{q}_{i}\sqrt{2\pi }}\text{exp}(\frac{-(y-{s}_{i}{)}^{2}}{2{q}_{{i}^{2}}} )$$

3 $$\sum_{i=1}^{k}{w}_{i}=1$$

where $k$ denotes the quantity and number of components, ${w}_{i}$ specifies the weight of the mixture component of *i*th element with the restraint $\sum_{i=1}^{k}{r}_{i}=1$. ${s}_{i} and {q}_{i}$ show the mean and variance of the *i*th component^24^.

### Steady-state probability of Markov chain

A discrete stochastic process (Markov chain) describes a possible sequence of events^38^. Markov chain models can be used to predict the probabilities of incoming process states. It plays an important role in projecting future droughts. To assess drought conditions of different climatic regions, several authors used Markov chain stochastic process models, including;^39–42^. The Markov chain, Transition Probability Matrix (TPM), and steady-state probabilities are explained briefly below:

Let Z = {${z}_{1 }, {z}_{2 } . . . . .{z}_{r}$} be the possible process states. The process may begin in one of these states and move sequentially from one to another state. If the current position of the chain is in the state ${z}_{i}$, then it proceeds to the next step, by passing to the state ${z}_{j}$ with probability ${P}_{ij}$. The TPM provides the probabilities of changing states^42^. TPM assumes that it is always in a square matrix, where rows show the initial state and columns show the next state. Each element of TPM is a probability, which means all values are nonnegative (0 ${\le P}_{ij }\le$ 1) and the sum of rows is equal to 1.

The following conditions satisfy each formulated TPM.

4 $${\sum P}_{ij}=1 \text{and }{P}_{ij}\le 1,$$

for all $i$ and $j$.

These probabilities are expressed in matrix form as follows.

Let ${Q}_{ij}^{(r)}$ be the number of transitions, in which ${z}_{i}$(initial state) transit to state ${z}_{j}$ (Next state). The different states of the transition probabilities are:

5 $${\sum P}_{ij}=\frac{{Q}_{ij}^{(r)}}{Qi}\text{ for }i, j = \text{1,2}\dots \dots \dots m$$

TPM is a square matrix with elements that are both real and non-negative, which are as follows:

6 $${P}_{ij}^{(n)}=\left[\begin{array}{c}{p}_{11} \cdots {p}_{1n}\\ \vdots \vdots \cdots \vdots \\ {p}_{n1 }\cdots {p}_{nm}\end{array}\right]$$

The above-mentioned matrix's elements assess the transient probabilities in the process state space. The stationary probabilities of the process quantify the long-term behavior of the process states. Such types of probabilities are known as steady-state probabilities. After a certain number of steps, a Markov process's probabilities tend towards a stable steady state. Let ${p}_{j}$ represent the limiting probability of ${i}^{th}$ step after “n” steps. The mathematical definition of the steady-state probability is defined below.

7 $$\sigma \left(j\right)=\text{lim}P\left({X}_{n}-j|{X}_{o}-i\right)$$

In another way,

8 $$\sigma \left(j\right)={}_{n\to \infty }{}^{lim}{P}_{ij}^{(n)}$$

The criteria by which MRPFE index values are categorized into different drought classes are provided in^24^.

### Mann–Kendall (MK) test

Mann–Kendall (MK) trend test has several applications in environmental and hydrological research and uses test statistics for assessing trends in time series data^43^. The MK trend test identifies statistically significant increasing or decreasing trends in long-term temporal data and detects climate trends in meteorological and hydrological time series data^44^. Several researchers have employed the MK test to identify trends, for example,^45^ used the modified MK test to detect trends in annual precipitation and temperature for nine states of the northeastern United States. The modified MK test is a statistical method that is built upon the original MK trend test by incorporating adjustments or enhancements to better suit specific research contexts.^46^ analyzed the long-term spatio-temporal variations in rainfall from 1901 to 2015 in India using the MK test to identify the pattern of precipitation (rainfall). Vicente-Serrano t al.^36^ employed the MK test to determine the monthly and annual patterns (trends) of the Yangtze River flows at the Zhutuo and Cuntan stations of China over 35 years (1980–2015). Praveen et al.^47^ employed the MK test to identify potential trends and analyze monthly and annual trends in streamflow, rainfall, and temperature within the Urmia Lake (UL) basin over 42 years from 1971 to 2013. The MK test can be mathematically described as follows:

9 $$\text{S}=\sum_{i=1}^{n-1}\sum_{j=k+1}^{n}sign\left({Y}_{j}-{Y}_{i}\right)$$

where $sign\left({\text{Y}}_{\text{j}}-{\text{Y}}_{\text{i}}\right)$ of Eq. (9) can be calculated by using Eq. (10).

10 $$sign\left({\text{Y}}_{\text{j}}-{\text{Y}}_{\text{i}}\right)=\left\{\begin{array}{c}+1\left({Y}_{j}-{Y}_{i}\right) >0\\ 0 \left({Y}_{j}-{Y}_{i}\right) =0\\ -1 \left({Y}_{j}-{Y}_{i}\right) <0\end{array}\right.$$

The positive S values represent an upward trend, negative values indicate a downward trend, and zero signifies the absence of a trend. The following test statistics are formulated to appraise trends within the complete time series data.

11 $${\text{Z}}_{\text{s}}=\left\{\begin{array}{l}\frac{S-1}{\sqrt{Var(S)}}\, if\, S>0 \\ 0\, if\, S=0\\ \frac{S+1}{\sqrt{Var(S)}}\, if\, S< 0\end{array}\right.$$

In Eq. (11), $Var(S)$ can be calculated by the following equation:

12 $$Var\left(s\right)=\frac{\mathbf{n}\left(\mathbf{n}-1\right)\left(2\mathbf{n}+5\right)-{\sum }_{\mathbf{i}=1}^{\mathbf{m}}{\mathbf{t}}_{\mathbf{j}}\left({\mathbf{t}}_{\mathbf{i}}-1\right)\left(2{\mathbf{t}}_{\mathbf{i}}+5\right)}{18}$$

where m is the difference in the number of compared values and n represents the overall amount of data points.

### Sen’s slope estimator (SSE)

Sen's Slope Estimator (SSE) serves as a nonparametric statistical test widely utilized for determining trend magnitudes in time series data^48^. SSE finds application in hydro-meteorological time series for both trend analysis^44^, and the prediction of trend magnitude^49,50^. SSE has been employed in several studies to gain insight into time series data trends. For example,^51^ examined spatiotemporal trends in annual rainfall utilizing SSE. Harka et al.^52^ calculated trends in the time series data of identified COVID-19 cases in India using SSE. Additional applications are found by^51,53–56^. Sen’s Slope Estimator (SSE) was introduced by^57^. A brief mathematical description of SSE is as follows:

13 $${J}_{i}=\frac{({Z}_{t}-{Z}_{i})}{t-1}\,for\, i=\text{1,2},\text{3,4}\dots \dots N$$

where ${Z}_{t}$ and ${Z}_{i}$ denote data values at times t and i, respectively in the context of t > i, ${J}_{i}$ signifies the slope of the estimator between the data points ${Z}_{t}$ and ${Z}_{i}$. Here $t$ varies from 2 to $n$ and $i$ varies from 1 to $N-1$, and n denotes the total number of data points in the temporal data.

For an individual datum in every period, there will be $N = n(n-1)/2$ slope estimates. For several observations in one or more periods, then $N\backslash n(n - 1)/2$.

The median of n values of ${Q}_{i}$ calculated by the following equation:

14 $${Q}_{i} = {Q}_{\left[\left(\frac{N+1}{2}\right)\right]} \text{\,if}\,n\, \text{is\, odd}$$

15 $${Q}_{i}=\left({Q}_{\left[\frac{N}{2}\right]}+{Q}_{\left[\frac{N+2}{2}\right]}\right)\text{if }n\text{ is even}$$

The Positive value of ${Q}_{i}$ indicate an upward (increasing) trend, while a negative value of ${Q}_{i}$ indicate a downward (decreasing) trend.

### Application

This research applies temporal data of precipitation from 18 climate models of CMIP6, emphasizing 32 grid points located on the Tibetan Plateau which national territory of China. However, we selected these models are grid points by following the study of^24^. Tibetan plateau encompasses an area of more than 2.5 million km^2^ (26.00–39.47 N, 73.19–104.47 E), this is the world’s largest plateau^58^. This region is also called the “world water tower”^59^. The Tibetan Plateau is a region in southwest China, and a large number of Asian rivers originate there. However, the region of the Tibetan Plateau is prone to global warming and climate change^60^. The temperature on the Tibetan Plateau has significantly increased over the last few decades^61^. So, it is beneficial to measure and assess drought with respect to global warming and climate change. Several researchers have performed spatial–temporal analyses associated with drought forecasting, assessment, and monitoring, in Tibetan Plateau regions. Including;^62–66^. In this study, we use simulated monthly time series of precipitation data of the CMIP6 models, which range from 1961 to 2014. We utilized CN05.1 model data as the observational data set of precipitation^32^. In addition, we utilize three different future scenarios i.e., SSP1-2.6, SSP2-4.5, and SSP5-8.5. Information on the selected models is available in^24^.

## The proposed method

**Figure 1:**
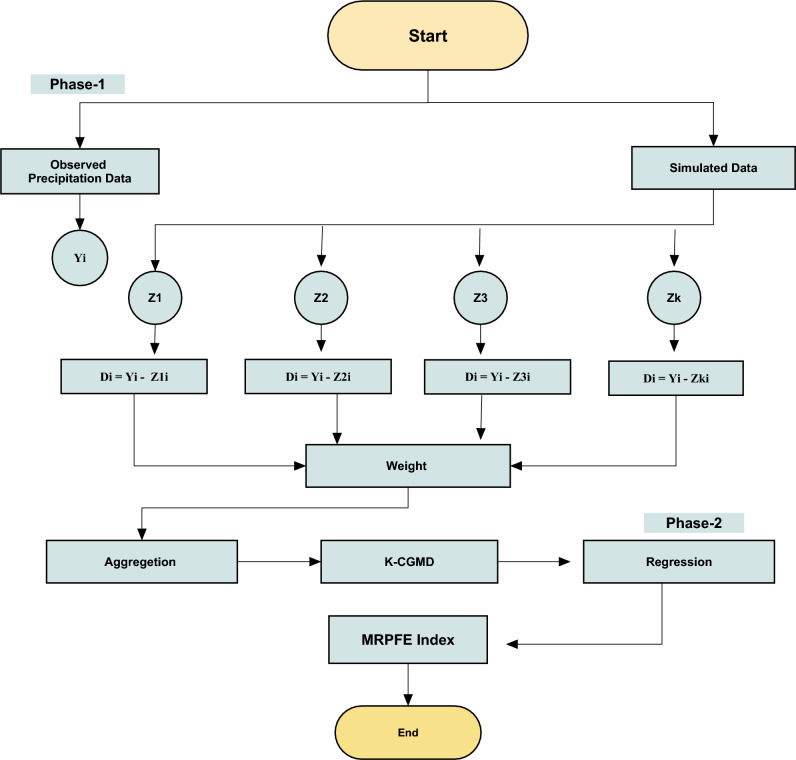
Flowchart of the proposed index.

In this section, we used precipitation data ranging from 1961 to 2014 from several GCMs corresponding to CN05.1 as observational data. Moreover, this section describes the process involved in the development of the MRPFE index. Figure 1 shows the flowchart of the MRPFE index. Here, the proposed weighting scheme aims to reduce the impact of extreme values on the aggregated data. The mathematical expressions for the suggested weighting scheme for combining precipitation time series data obtained from various CMIP6 models are shown in this section. The proposed weighting scheme distinguishes itself by giving more weight to those values whose frequency contributes more to homogeneity among them. In contrast, divergent values will be given lower weights. This implicates minimization of the impact of extreme values in the aggregation process. The proposed weighting scheme is implemented in the following steps:

Let D $\in$ (${\text{Z}}_{1}$,${\text{ Z}}_{2}$, ${\text{Z}}_{3}$,….${\text{Z}}_{\text{K}}$) be the time series data of precipitation simulated by various models in a specific region. And $y$ is the observed time series data. Where $K$ shows the total number of GCMs. The primary goal of the weighting scheme is to reduce the effect of extreme values. Our proposed scheme is based on three major phases. The explanation of each phase is described below:

Phase 1. Weighting each model

This phase assigns each model weight based on its difference from observed values. Below is a description of these steps:

Step 1. The absolute difference between simulated and observed

In this stage, we are taking the absolute difference of data simulated from GCM models $({Z}_{i})$ and observed data $y$:

16 $$a1 = \left| { y {-} Z_{i} } \right|$$

Step 2. Combining observed and simulated data

In this stage, we are adding the absolute of $y$ to the absolute of each model value:

17 $$a2= |y| + |{Z}_{i}$$

Step 3. Assigning weights

This stage assigns weights to each model by taking the ratio of Eqs. (16) and (17):

18 $$T = \frac{a1}{a2}$$

Here, ${T}_{i}$ will be the weight of *i*th model.

Step 4. Standardization of weights

This stage standardizes the weights assigned to each model. For standardization, each assigned weight is divided by the sum of the weights of each model:

19 $${S}_{i}=\frac{{T}_{i}}{\sum {T}_{i}}$$

Here ${S}_{i}$ represents the standardized weights for each model.

Phase 2. Assigning spatiotemporal weights to each value

This phase assigns values of each model a relevant weight based on its location and time. This phase includes the following steps:

Step 1. Exponentials of the absolute differences

As a_1_ in Eq. (16) represents the difference in the observed value and *i*th GCM data, the first step of this phase then suggests calculating the exponent of *i*th differences. This equation aims to maximize the differences.

20 $${j}_{i}= {e}^{{a}_{1}}$$

Step 2. Estimation of weights

In this step, we assign a high weight to small deviated values and a low weight to large deviated values:

21 $${p}_{i}=1- \frac{{j}_{i}}{\sum_{i}^{k}{j}_{i}}$$

Step 3. Standardizing weights

In this stage, we standardize ${p}_{i ,}$ as follows:

22 $${w}_{i}=\frac{{p}_{i}}{\sum_{i=1}^{K}{p}_{i}}$$

Under certain conditions that ${\sum }_{i=1}^{K} {w}_{i}=1.$ Phase 2 is iterated for each model.

Phase 3. Hybridization of Phases 1 & 2

To combine both weights, we are taking an average of Eqs. (19) and (22):

23 $${P}_{i}=\frac{{w}_{i}+{S}_{i}}{2}$$

Here ${P}_{i}$ are representing the proposed weights and we name this weighting technique “Weighted Ensemble”.

Phase 4. Data aggregation

This phase aggregates the data of various CMIP6 GCM simulations under proposed weights:

24 $${P}_{{\varvec{c}}{\varvec{t}}}=\sum_{i=1}^{k}{Z}_{i}{P}_{i}$$

After the aggregation of data, now we perform multiple linear regression models for future projections.

After this, we will standardize the ${P}_{{\varvec{c}}{\varvec{t}}}$ under K-CGMD, this is the 12-component combined Cumulative Distribution Function (CDF):

25 $$M\left(x\right) = M{(P}_{{\varvec{c}}{\varvec{t}}1\boldsymbol{ }})+ M{(P}_{{\varvec{c}}{\varvec{t}}\boldsymbol{ }2})+ M{(P}_{{\varvec{c}}{\varvec{t}}\boldsymbol{ }3})\dots . M{(P}_{{\varvec{c}}{\varvec{t}}\boldsymbol{ }12})$$

Here, we selected 12 components as there are 12 months in a year. In Eq. 25, $M(x)$ is the CDF of K-CGMD. To standardize this CDF for the calculation of the proposed drought index MRPFRE, the following method is applied:

26 $$MRPFE = h ( n- \frac{{C}_{o}+ {C}_{1}tn+{C}_{2}{n}^{2} }{1+{q}_{1}n + {q}_{2}{n}^{2}+ {q}_{3}{n}^{3}} )$$

27 $$n= \sqrt{\text{ln}(\frac{1}{{\left\{M(X)\right\}}^{2}})}$$

Here $$h= -1$$, when

28 $$0 \le M(x)\le 0.5$$

29 $$MRPFE = h ( n- \frac{{C}_{o}+ {C}_{1}n+{C}_{2}{n}^{2} }{1+{q}_{1}n + {q}_{2}{n}^{2}+ {q}_{3}{n}^{3}} )$$

where

30 $$n= \sqrt{\text{ln}(\frac{1}{{\left\{1- M(X)\right\}}^{2}})}$$

Here $$h= +1$$, when

31 $$0.5 \le M(x) \le 1$$

${\text{C}}_{o}$ = 2.515517, ${\text{C}}_{1}$ = 0.802853, ${\text{C}}_{2}$ = 0.010328, ${\text{q}}_{1}$ = 1.432788,${\text{ q}}_{2}$ = 0.985269, and ${\text{q}}_{3}$ = 0.001308 are constants. This index and the included constants were developed based on the spatiotemporally weighted combination of precipitation time series. It is named the Maximum Relevant Prior Feature Ensemble (MRPFE) index.

## Comparative statistics

### Simple model averaging (SMA)

Simple model averaging (SMA) is a type of simple mean that gives equal importance to each value in the dataset^67^, which has been used many times to combine GCM ensembles^29,68^. In this study, it is applied as a comparative method to the proposed weighting scheme. The calculation is based on the following equation:

32 $$S \left(t\right)=\frac{1}{k} \sum_{i=1}^{k}{\widehat{P}}_{i}$$

where $S(t)$ is the SMA of GCMs, ${\widehat{P}}_{i}$ is the precipitation projection for the *i*th GCM and $k$ is the number of GCMs.

### Relative absolute error (RAE) and mean absolute error (MAE)

Relative Absolute Error (RAE), a statistical tool, assesses the accuracy and precision of projections relative to a reference value. RAE is calculated by dividing the absolute difference between the predicted and the reference value. In contrast, MAE, another statistical performance metric, represents the average of the absolute differences between predicted and corresponding reference values. These methods are frequently used in various recent studies^69^.^24,69^ have explained these methods mathematically in their studies.

## Results

### Estimation of weights of the proposed index

**Figure 2:**
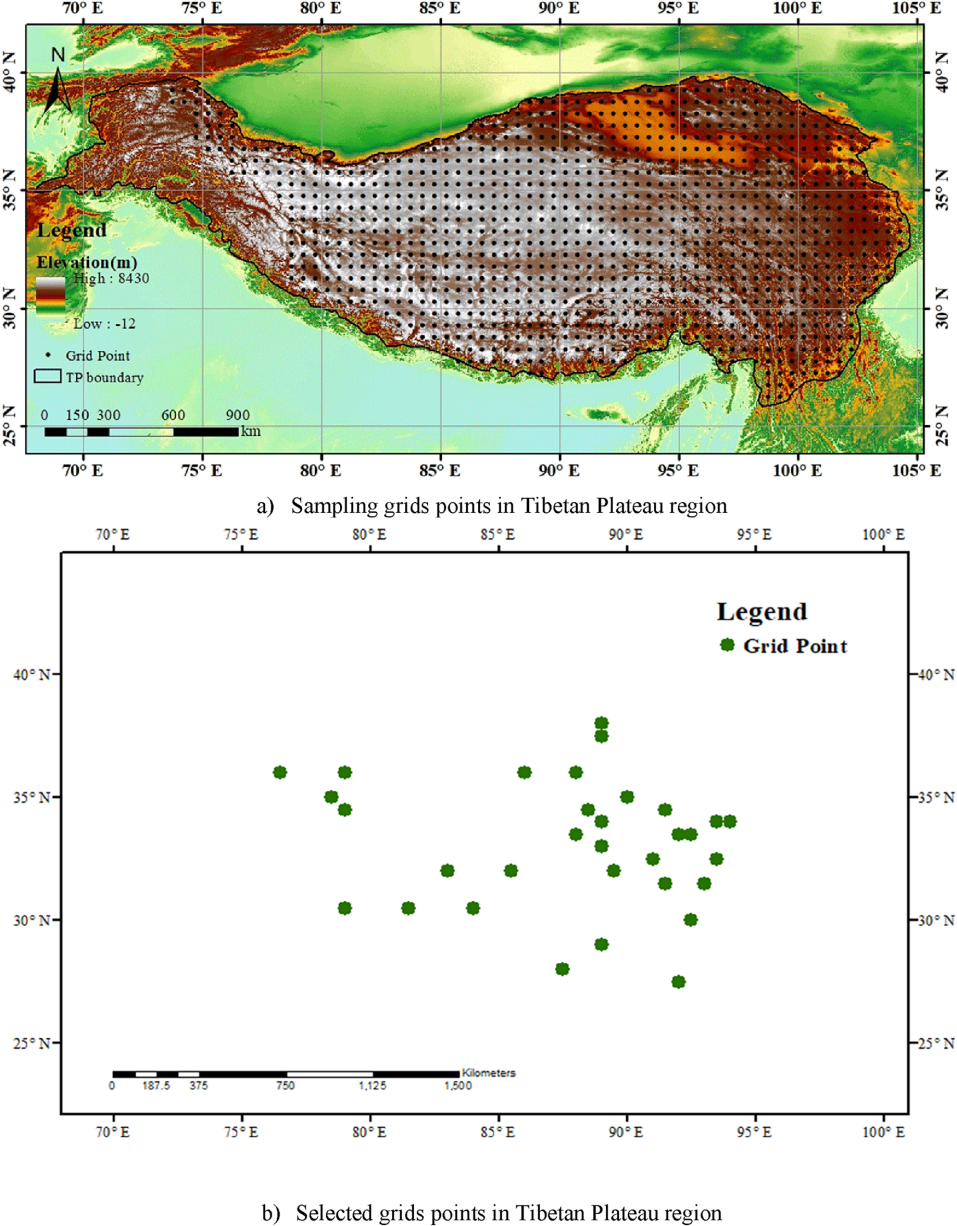
The geographical locations of the selected study area.

**Figure 3:**
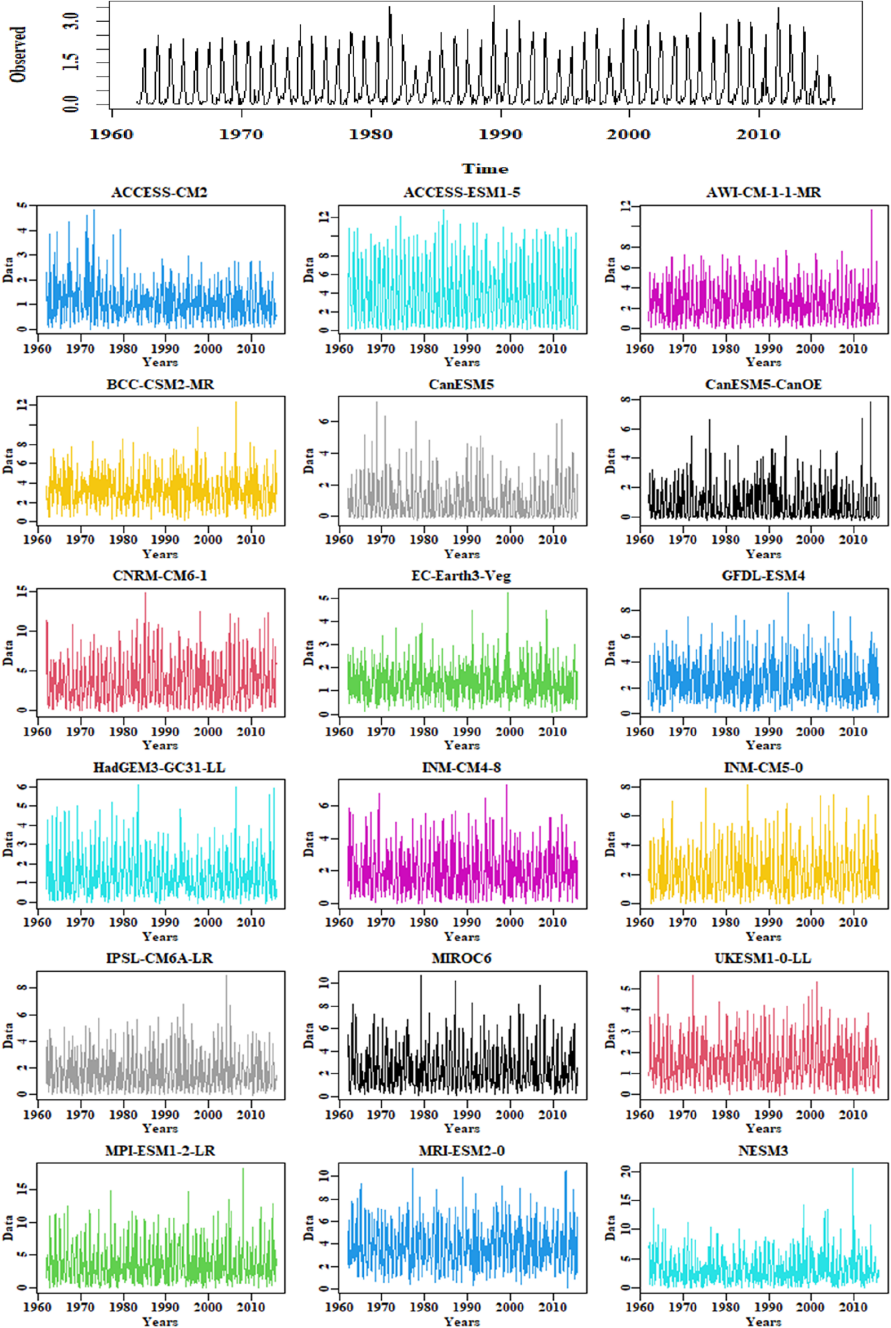
Temporal representation of observed and selected GCM data of precipitation (kgms^−2^).

**Figure 4:**
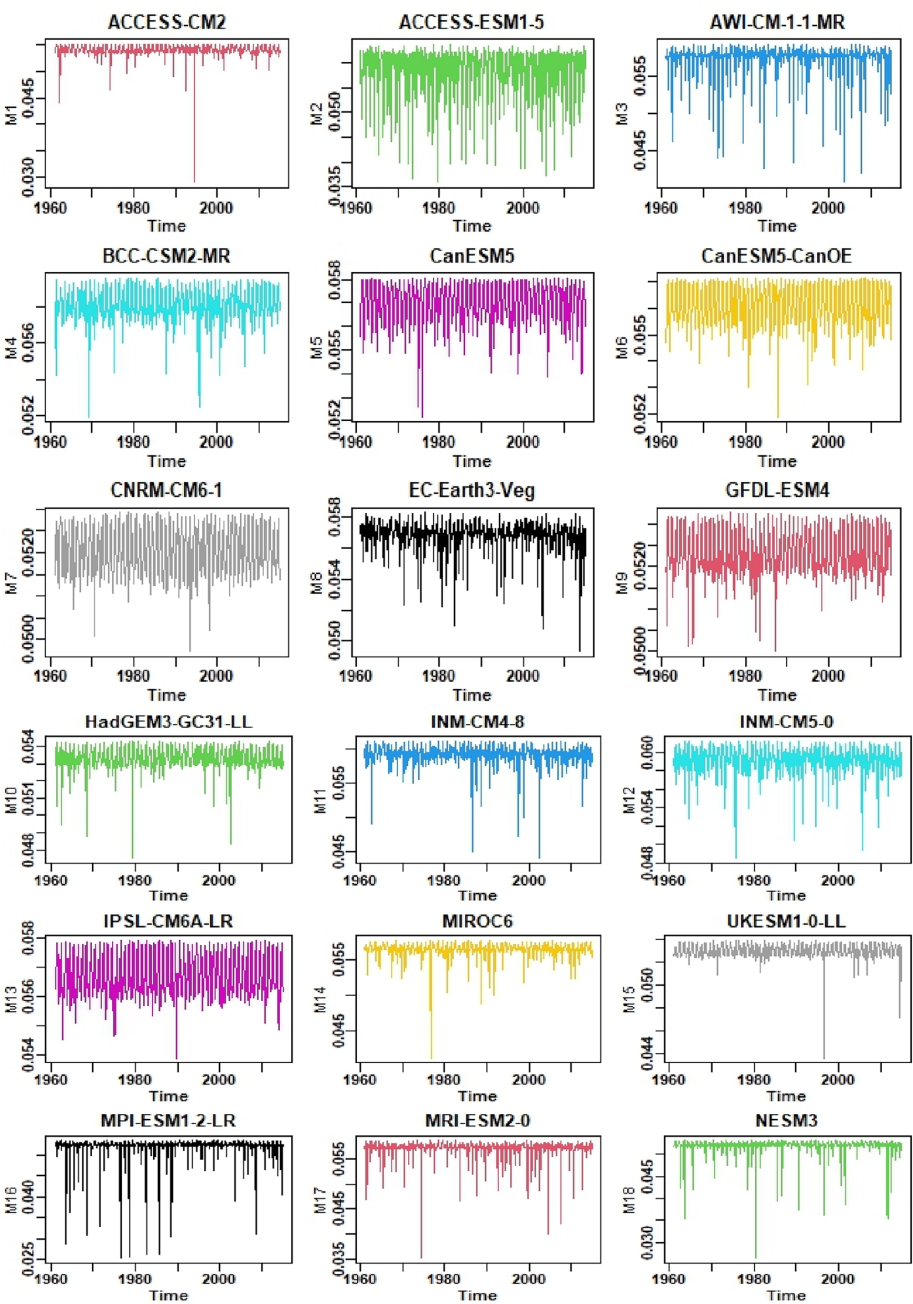
Weights assigned to each model at one random location.

In this study, a novel weighting index is proposed to address biases and reduce the impact of extreme precipitation values by placing greater emphasis on values that deviate less from observational data. Figure 2 presents the selected locations of the Tibetan Plateau. A temporal representation of observed and simulated models data is shown in Fig. 3. Table 1 provides the resolution of each selected model and summary statistics of weights assigned to all selected GCMs at one random point. The GCMs and their corresponding weights are listed in rows, and the columns show the minimum, maximum, and average weights assigned to each GCM. It can be observed that the average weights assigned to the GCMs range from 0.046 to 0.054. The minimum and maximum weights assigned to each GCM also vary, with some GCMs having weights as low as 0.023 and as high as 0.057. The maximum average weight (0.054) is assigned to MPI-ESM1-2-LR, minimum average weight (0.046) to CNRM-CM6-1. Overall, the table provides useful information about the weights assigned to the GCMs and their relative importance in the ensemble. Furthermore, Fig. 4 displays the assigned weights of each CMIP6 model at one random grid point. Table 2 shows the monthly weights that are assigned to all selected GCMs at one random point. From the table, we can see that the maximum weights were assigned to the ACCESS-ESM1-5 model in December and, the minimum to the MPI-ESM1-2-LR model in July.

**Table 1:** Resolution of models and summary statistics of weights assigned to each GCM at one random point.

Sr. No.	Models	Resolution (longitude × latitude)	Minimum	Maximum	Average
1	ACCESS-CM2	1.875° × 1.25°	0.0261	0.0491	0.0483
2	ACCESS-ESM1-5	1.875° × 1.2143°	0.0318	0.0567	0.0524
3	AWI-CM-1–1-MR	0.9375° × 0.9375°	0.0374	0.0526	0.0512
4	BCC-CSM2-MR	1.125° × 1.125°	0.0467	0.0529	0.0519
5	CanESM5	2.8125° × 2.8125°	0.0470	0.0516	0.0508
6	CanESM5-CanOE	2.8125° × 2.8125°	0.0466	0.0508	0.0500
7	CNRM-CM6-1	1.40625° × 1.40625°	0.0449	0.0471	0.0464
8	EC-Earth3-Veg	0.703125° × 0.703125°	0.0463	0.0518	0.0508
9	GFDL-ESM4	1.25° × 1°	0.0300	0.0554	0.0523
10	HadGEM3-GC31-LL	2° × 2.25°	0.0447	0.0474	0.0467
11	INM-CM4-8	2° × 1.5°	0.0427	0.0484	0.0476
12	INM-CM5-0	2° × 1.5°	0.0419	0.0544	0.0530
13	IPSL-CM6A-LR	2.5° × 1.25874°	0.0473	0.0545	0.0530
14	MIROC6	1.40625° × 1.40625°	0.0483	0.0515	0.0506
15	UKESM1-0-LL	0.9375° × 0.9375°	0.0371	0.0514	0.0505
16	MPI-ESM1-2-LR	1.875° × 1.875°	0.0430	0.0555	0.0537
17	MRI-ESM2-0	1.125° × 1.125°	0.0435	0.0477	0.0470
18	NESM3	1.875° × 1.875°	0.0235	0.0476	0.0465
		0.703125° × 0.703125°

**Table 2:** Monthly summary statistics of weights of all selected GCMs at one random point.

Sr. No.	Models	Jan	Feb	Mar	Apr	May	Jun	Jul	Aug	Sep	Oct	Nov	Dec
1	ACCESS-CM2	0.054	0.054	0.054	0.054	0.054	0.053	0.055	0.055	0.055	0.054	0.054	0.054
2	ACCESS-ESM1-5	0.062	0.061	0.059	0.056	0.052	0.049	0.060	0.060	0.059	0.057	0.061	0.062
3	AWI-CM-1-1-MR	0.058	0.058	0.058	0.057	0.058	0.057	0.055	0.055	0.057	0.058	0.058	0.058
4	BCC-CSM2-MR	0.058	0.058	0.058	0.058	0.058	0.059	0.059	0.059	0.058	0.058	0.058	0.058
5	CanESM5	0.056	0.056	0.057	0.057	0.058	0.058	0.058	0.058	0.058	0.057	0.056	0.056
6	CanESM5-CanOE	0.055	0.056	0.056	0.056	0.057	0.057	0.057	0.057	0.057	0.056	0.055	0.055
7	CNRM-CM6-1	0.051	0.052	0.052	0.052	0.052	0.053	0.053	0.053	0.052	0.052	0.052	0.051
8	EC-Earth3-Veg	0.057	0.057	0.057	0.057	0.057	0.057	0.057	0.057	0.055	0.057	0.057	0.057
9	GFDL-ESM4	0.052	0.052	0.052	0.052	0.052	0.053	0.053	0.053	0.053	0.052	0.052	0.052
10	HadGEM3-GC31-LL	0.053	0.053	0.053	0.053	0.054	0.053	0.054	0.054	0.054	0.053	0.053	0.053
11	INM-CM4-8	0.059	0.059	0.059	0.059	0.059	0.060	0.059	0.059	0.058	0.059	0.059	0.059
12	INM-CM5-0	0.059	0.059	0.058	0.058	0.059	0.060	0.060	0.059	0.059	0.060	0.060	0.059
13	IPSL-CM6A-LR	0.056	0.056	0.056	0.057	0.057	0.057	0.058	0.058	0.057	0.056	0.056	0.056
14	MIROC6	0.056	0.056	0.057	0.057	0.057	0.057	0.057	0.057	0.055	0.056	0.056	0.056
15	UKESM1-0-LL	0.053	0.053	0.053	0.053	0.053	0.053	0.054	0.053	0.053	0.053	0.053	0.053
16	MPI-ESM1-2-LR	0.052	0.052	0.053	0.053	0.053	0.053	0.048	0.049	0.052	0.052	0.052	0.052
17	MRI-ESM2-0	0.057	0.057	0.057	0.058	0.058	0.058	0.055	0.055	0.057	0.058	0.057	0.057
18	NESM3	0.052	0.052	0.052	0.053	0.053	0.053	0.049	0.050	0.052	0.052	0.052	0.052

### Validation of the proposed WE scheme

Table 3 shows the summary statistics of MAE and RAE of the WE weighting scheme and SMA technique. The results show that the errors of the WE scheme are significantly less than SMA scheme. Based on these findings, it can be stated that our proposed weighting scheme is more efficient than the traditional SMA scheme.

**Table 3:** Summary statistics of RAE and mean absolute error.

	SSP1-2.6		SSP2-4.5		SSP5-8.5	
Time scale	Unimodal	K-CGMD	Unimodal	K-CGMD	Unimodal	K-CGMD
1	− 222.27 (Skewed Normal)	− 742.13	− 158.65 (Skewed-normal)	− 743.43	− 129.03 (Skewed-normal)	− 840.03
3	− 323.31 (GEV)	− 2355.23	− 291.91 (Triangular)	− 2346.92	− 283.19 (Triangular)	− 2559.56
6	− 318.68 (GEV)	− 3139.93	− 300.10 (GEV)	− 3096.39	− 252.11 (GEV)	− 3325.64
9	-315.88 (Logistic)	− 3232.43	− 301.87 (Normal)	− 3043.03	− 255.50 (Logistic)	− 3293.38
12	− 353.10 (Laplace)	− 3276.15	− 263.96 (Logistic)	− 2972.94	− 297.35 (Johnson SU)	− 3194.17
24	− 524.45 (Johnson SU)	− 3921.03	− 381.67 (Normal)	− 3579.21	− 449.68 (3P Weibull)	− 3771.39
48	− 355.58 (Johnson SU)	− 4492.90	− 556.60 (Gumbel)	− 4112.98	− 381.56 (3P Weibull)	− 4418.03

### Estimation of MRPFE using K-CGMD under a different scenario

**Figure 5:**
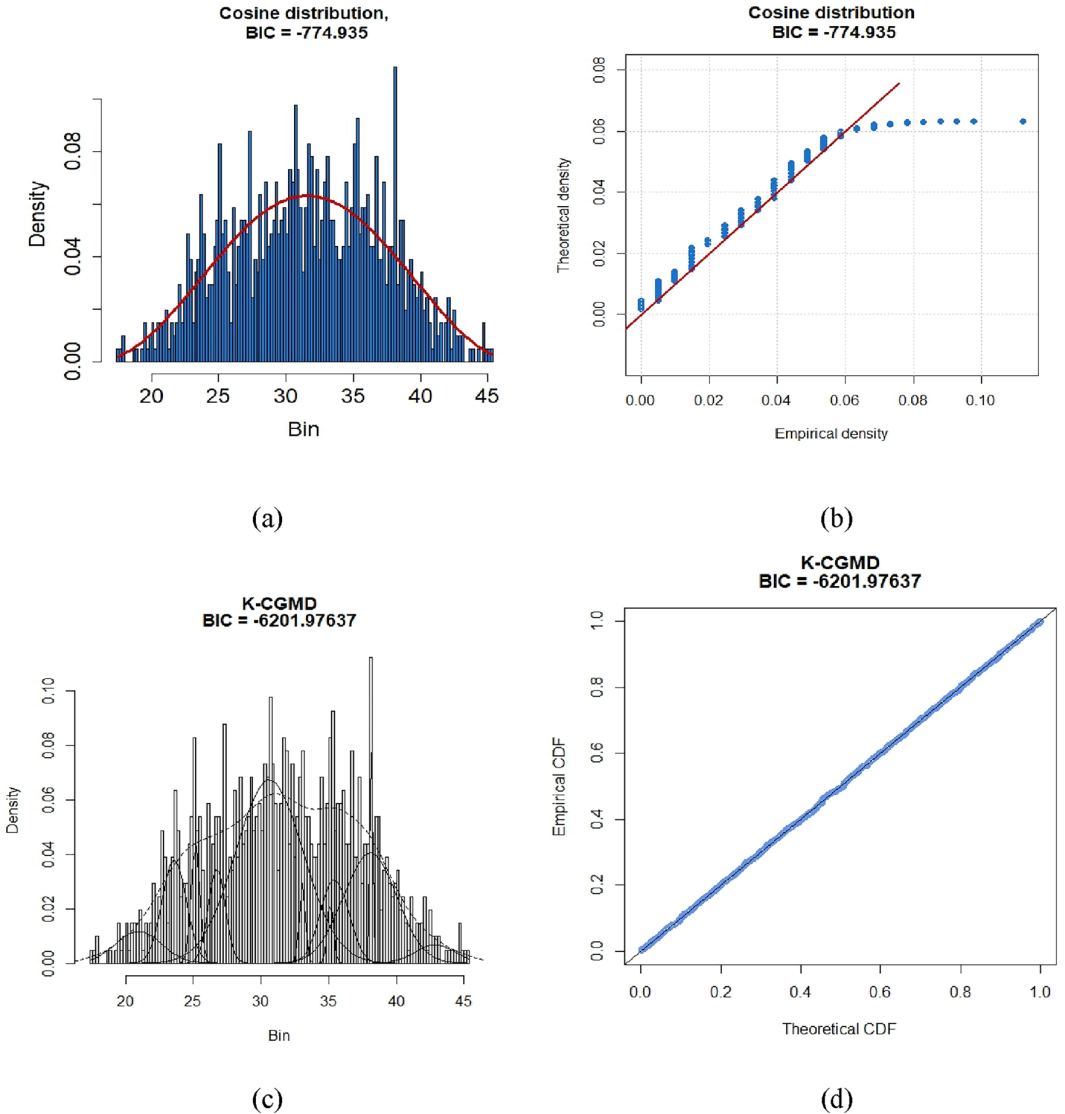
Probability and QQ-plot of univariate and K-CGMM for SSP1-2.6 future scenarios at time scale-1 on 76.5° E and 36° N location.

**Figure 6:**
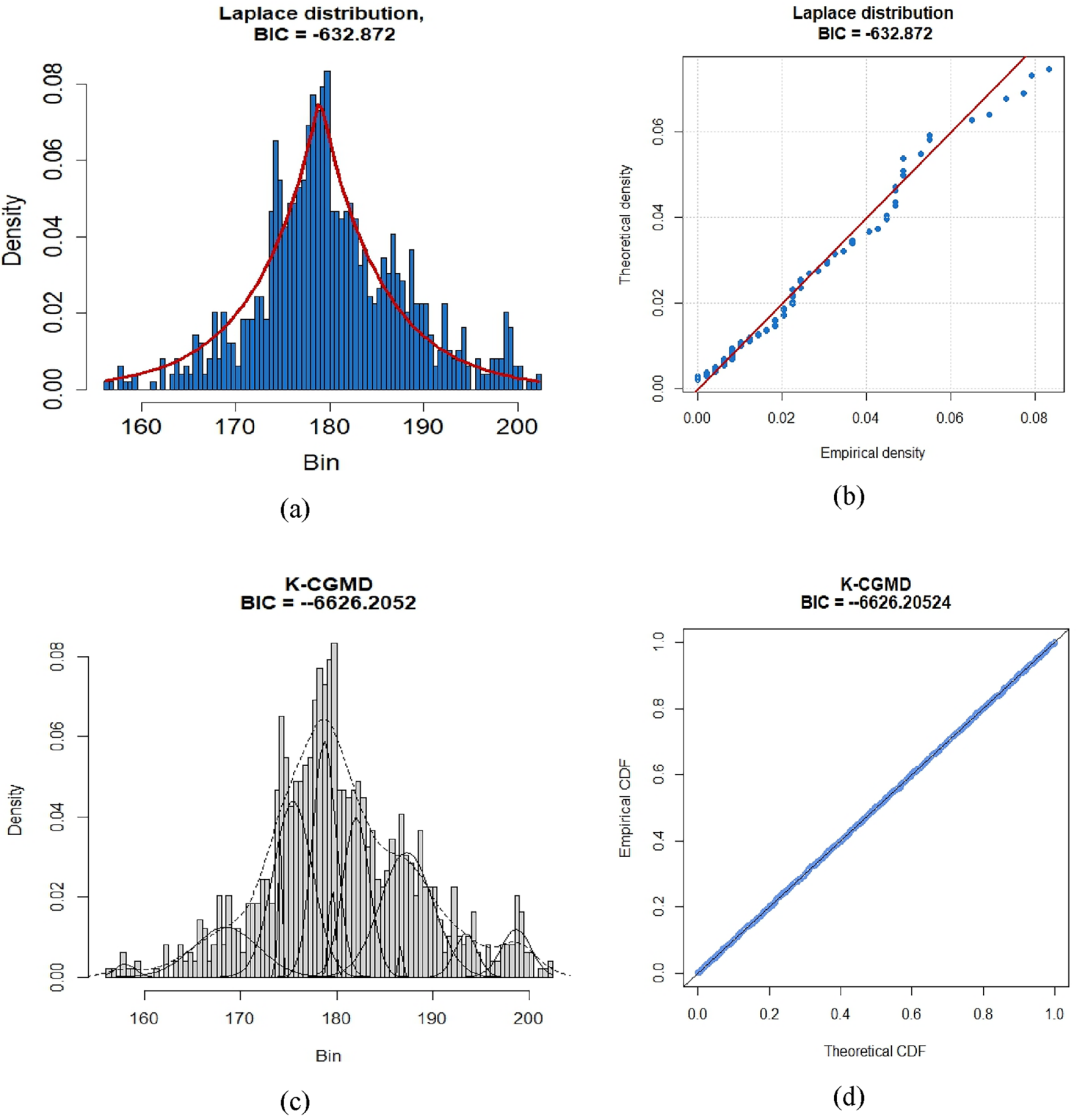
Probability and QQ-plot of univariate and K-CGMM for SSP2-4.5 future scenarios at time scale-1 on 76.5° E and 36° N location.

**Figure 7:**
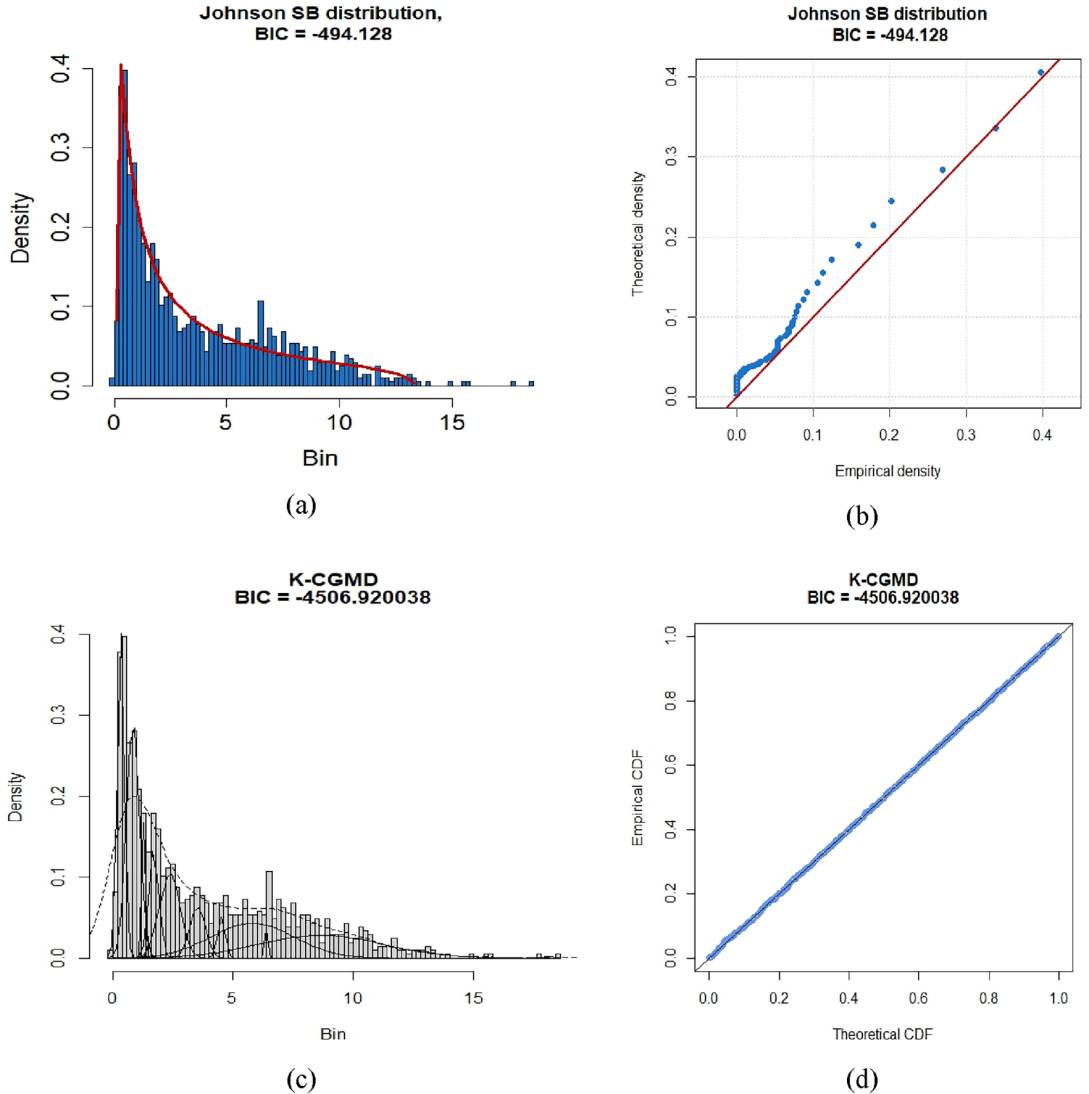
Probability and QQ-plot of univariate and K-CGMM for SSP5-8.5 future scenarios at time scale-1 on 76.5° E and 36° N location.

In this section, we examine the effectiveness of K-CGMD for modeling drought index values. The accuracy of K-CGMD at different time scales is compared with different univariate probability distributions using the Bayesian Information Criterion (BIC). BIC is used to evaluate models and determine which trade-off between model fit and complexity is optimal. A more favorable model fit is indicated by lower BIC values. Table 4 represents the BIC values for univariate distributions and the K-CGMD model for various time scales of three different scenarios. The findings reveal that, for SSP1-2.6, SSP2-4.5, and SSP5-8.5, the K-CGMD model consistently exhibits lower BIC values across all time scales compared to unimodal. This consistent pattern recommends that the K-CGMD model is a superior fit for the data for most of the time scales within all three scenarios. Consequently, the K-CGMD model proves to be a more reliable and effective approach for standardizing drought indices compared to unimodal distributions. In Fig. 5, probability and Q-Q plots visually demonstrate K-CGMD's superiority over unimodal probability models in modeling drought indices for scenario SSP1-2.6. Furthermore, Figs. 6 and 7 present probability and Q-Q plots for scenarios SSP1-2.6, SSP2-4.5, and SSP5-8.5, respectively, providing additional evidence of K-CGMD's superior fit for the data.

**Table 4:** BIC of Unimodal and K-CGMD at different time scales for three different scenarios at one location.

Metrics	Proposed			SMA		
	Minimum	Average	Maximum	Minimum	Average	Maximum
RAE	0.4500	2.8892	22.7670	0.4650	2.9710	23.6250
MAE	0.411	1.629	6.575	0.439	1.666	6.804

### Trend assessment under Mann–Kendall and Sen’s slope

The significance of future drought trends and direction is evaluated using Sen’s slope and seasonal Mann–Kendall approaches^36^. This specific method is used to evaluate the drought conditions on the Tibetan Plateau. Table 5 presents trend analyses utilizing Mann–Kendall and Sen’s slope methods for three scenarios across various time scales. Each scenario and time scale combination includes the Kendall Z-value, Sen’s slope, *p*-value, significance level, and trend direction. The direction of the trend, whether it is increasing or decreasing, is determined by the trend analysis. Additionally, the *p*-value i.e., *p* < 0.05 establishes the significance level of the trend in this analysis. The results indicate a predominantly decreasing trend across all time scales for SSP1-2.6, with an exception at time scale 1, which exhibits an increasing trend. For SSP2-4.5, the trend is mostly increasing for smaller time scales (Scale-1, Scale-3, and Scale-6) and decreasing for larger time scales (Scale-24 and Scale-48). For SSP5-8.5, the trend is consistently decreasing for all time scales and is statistically significant for all except for Scale-1.

**Table 5:** Mann–Kendall and Sen’s Slope values for trend evaluation under three different future scenarios at each time scale.

Scenarios	Time Scales	z-value	Slope	*p*-value	Significance	Direction
SSP1-2.6	1	0.109	0.000	0.913	*p* > 0.10	Increasing
	3	− 0.105	0.000	0.917	*p* > 0.10	Decreasing
	6	− 1.392	− 0.001	0.164	*p* > 0.10	Decreasing
	9	− 2.332	− 0.003	0.020	*p* < 0.05	Decreasing
	12	− 2.304	− 0.003	0.021	*p* < 0.05	Decreasing
	24	− 2.827	− 0.004	0.005	*p* < 0.05	Decreasing
	48	− 6.008	− 0.009	0.000	*p* < 0.05	Decreasing
SSP2-4.5	1	0.169	0.000	0.866	*p* > 0.10	Increasing
	3	0.550	0.000	0.583	*p* > 0.10	Increasing
	6	0.137	0.000	0.891	*p* > 0.10	Increasing
	9	− 0.404	0.000	0.686	*p* > 0.10	Decreasing
	12	− 1.033	− 0.001	0.302	*p* > 0.10	Decreasing
	24	− 2.511	− 0.003	0.012	*p* < 0.05	Decreasing
	48	− 6.144	− 0.009	0.000	*p* < 0.05	Decreasing
SSP5-8.5	1	− 1.582	− 0.002	0.114	*p* > 0.10	Decreasing
	3	− 3.247	− 0.003	0.001	*p* < 0.05	Decreasing
	6	− 5.366	− 0.005	0.000	*p* < 0.05	Decreasing
	9	− 7.410	− 0.008	0.000	*p* < 0.05	Decreasing
	12	− 8.496	− 0.012	0.000	*p* < 0.05	Decreasing
	24	− 11.537	− 0.016	0.000	*p* < 0.05	Decreasing
	48	− 14.593	− 0.020	0.000	*p* < 0.05	Decreasing

### Estimation of drought using steady-state probabilities

The steady-state probability of the Markov chain is employed in this study to measure the long-term impact of random events. Precipitation is classified into seven classes, namely Extreme Wet (EW), Severe Wet (SW), Moderate Wet (MW), Near Normal (NN), Moderate Drought (MD), Severe Drought (SD), and Extreme Drought (ED). Table 6, evaluates the steady-state probabilities for the various drought classes under three different scenarios at each time scale. From this information, we conclude that the probability of ED is greater than the probability of EW the probability of MD is less than the probability of MW, and the probabilities SD is greater than the probabilities of SW at SSP1-2.6. After analyzing the probabilities of SSP2-4.5 and SSP5-8.5 across different time scales we noticed that the probability of dry conditions is more probable than that of wet conditions. It is anticipated that in all locations, drought conditions will be more prevalent than wet conditions.

**Table 6:** Steady State Probabilities for various drought classes in various time scales at all the locations by using three different scenarios.

Scenario	Time scales	ED	EW	MD	MW	NN	SD	SW
SSP1-2.6	1	0.0156	0.0019	0.0873	0.0989	0.6702	0.0641	0.0621
	3	0.0293	0.0155	0.0802	0.1047	0.6710	0.0411	0.0582
	6	0.0234	0.0234	0.0897	0.0858	0.6803	0.0478	0.0497
	9	0.0247	0.0234	0.0881	0.0867	0.6827	0.0438	0.0506
	12	0.0207	0.0205	0.0985	0.0840	0.6728	0.0468	0.0566
	24	0.0216	0.0236	0.0996	0.0777	0.6809	0.0473	0.0492
	48	0.0224	0.0285	0.0844	0.1037	0.6829	0.0478	0.0305
SSP2-4.5	1	0.0204	0.0116	0.0951	0.0912	0.6906	0.0388	0.0524
	3	0.0194	0.0243	0.1079	0.0914	0.6676	0.0418	0.0476
	6	0.0224	0.0185	0.1023	0.0965	0.6676	0.0448	0.0478
	9	0.0176	0.0215	0.0792	0.0919	0.6892	0.0567	0.0440
	12	0.0206	0.0225	0.0990	0.0922	0.6775	0.0431	0.0451
	24	0.0227	0.0243	0.0770	0.0936	0.6914	0.0464	0.0445
	48	0.0246	0.0225	0.0984	0.1012	0.6763	0.0359	0.0411
SSP5-8.5	1	0.0310	0.0155	0.1018	0.0824	0.6857	0.0252	0.0582
	3	0.0204	0.0253	0.0943	0.0865	0.6851	0.0447	0.0437
	6	0.0224	0.0283	0.0906	0.0838	0.6930	0.0458	0.0361
	9	0.0244	0.0235	0.0978	0.0880	0.6794	0.0420	0.0450
	12	0.0161	0.0224	0.0837	0.0849	0.6829	0.0612	0.0488
	24	0.0238	0.0185	0.0928	0.0876	0.6686	0.0571	0.0516
	48	0.0295	0.0250	0.1005	0.0872	0.6832	0.0404	0.0341

## Discussion

The analysis of the proposed MRPFE drought index and the novel weighting scheme WE demonstrate significant improvements in the accuracy of drought estimation. The application of the WE scheme to the CMIP6 dataset shows a marked reduction in both comparative measures compared to the SMA approach.

CMIP models have various uncertainties and this unpredictability is an essential part of climate modeling. Understanding these uncertainties is integral for making informed decisions regarding climate change mitigation. By utilizing MME, probabilistic approaches, and transparent communication, we can better manage uncertainties and enhance the robustness of climate projections. Variations in the analysis results of drought trends under different scenarios arise from the varying assumptions and projections related to future greenhouse gas emissions, land use changes, and other socio-economic factors. Each scenario reflects a different trajectory of human activity and its impact on the climate system, leading to variations in the projected severity, frequency, and spatial distribution of droughts. And the guiding significance of utilizing different emission scenarios is to help policymakers and to get insight into better risk management. The proposed index employs the K-CGMD and Markov Chain steady-state probability analysis, provides a robust framework for estimating the likelihood of various drought states. The results indicate that the MRPFE index effectively captures the temporal dynamics of drought conditions, offering a more nuanced understanding of drought trends in this region. Furthermore, the study’s findings highlight the importance of considering multiple emission scenarios when projecting future drought conditions. The variations in drought trends observed under different scenarios underscore the influence of future greenhouse gas emissions, land use changes, and other socio-economic factors on drought severity, frequency, and spatial distribution. This insight is crucial for policymakers and stakeholders involved in climate change mitigation and adaptation planning.

## Conclusion

Drought is a naturally occurring phenomenon, that is caused by irregularities in climate variables such as precipitation patterns. There are numerous ecological causes concerned with classifying drought conditions at the particular monitoring station. Therefore, proper pattern processing methods are required to project and investigate the periodic data about the occurrences of drought classes. This study provides a novel weighting scheme, “WE” to combine multiple models and a new drought index “MRPFE” to project drought. The novel weighting scheme WE utilize time-series precipitation data from various GCMs at a specific georeferenced point. In application, simulated time-series precipitation data of CMIP6 from 18 GCMs at thirty-two random locations of the Tibetan Plateau region of China has been used. The methodology of the MRPFE drought index is based on K-CGMD. To estimate the probability of specific drought states, the study includes a steady-state probability analysis applying the Markov Chain approach. The MAE and RAE have been used as relative measures to assess the performance of the proposed weighting scheme. The comparative inference shows that the proposed weighting scheme has greater efficiency than SMA in combining GCMs. Looking ahead, the findings suggest that the Tibetan Plateau region may experience increase in frequency of drought due to declining pattern of precipitation.

## Data Availability

The data and code that support the findings of this study are available from the corresponding author upon reasonable request. Declaration of generative AI and AI-assisted technologies in the writing process. During the preparation of this work the authors used ChatGBT in order to improve the readability of the article. This tool was used in the review and editing stages of drafts only. After using this tool, the authors reviewed and edited the content as needed and take full responsibility for the content of the publication.
